# A Distributed Parallel Algorithm Based on Low-Rank and Sparse Representation for Anomaly Detection in Hyperspectral Images

**DOI:** 10.3390/s18113627

**Published:** 2018-10-25

**Authors:** Yi Zhang, Zebin Wu, Jin Sun, Yan Zhang, Yaoqin Zhu, Jun Liu, Qitao Zang, Antonio Plaza

**Affiliations:** 1 School of Computer Science and Engineering, Nanjing University of Science and Technology, Nanjing 210094, China; yzhang@njust.edu.cn (Y.Z.); sunj@njust.edu.cn (J.S.); zhuyaoqin@163.com (Y.Z.); liuj302@126.com (J.L.); zangqitao@163.com (Q.Z.); 2 Lianyungang E-Port Information Development Co. Ltd., Lianyungang 222042, China; yzhang@lygeport.gov.cn; 3 Hyperspectral Computing Laboratory, Department of Technology of Computers and Communications, University of Extremadura, 10003 Caceres, Spain; aplaza@unex.es

**Keywords:** hyperspectral images, anomaly detection, distributed and parallel computing, apache spark, clouds

## Abstract

Anomaly detection aims to separate anomalous pixels from the background, and has become an important application of remotely sensed hyperspectral image processing. Anomaly detection methods based on low-rank and sparse representation (LRASR) can accurately detect anomalous pixels. However, with the significant volume increase of hyperspectral image repositories, such techniques consume a significant amount of time (mainly due to the massive amount of matrix computations involved). In this paper, we propose a novel distributed parallel algorithm (DPA) by redesigning key operators of LRASR in terms of MapReduce model to accelerate LRASR on cloud computing architectures. Independent computation operators are explored and executed in parallel on Spark. Specifically, we reconstitute the hyperspectral images in an appropriate format for efficient DPA processing, design the optimized storage strategy, and develop a pre-merge mechanism to reduce data transmission. Besides, a repartitioning policy is also proposed to improve DPA’s efficiency. Our experimental results demonstrate that the newly developed DPA achieves very high speedups when accelerating LRASR, in addition to maintaining similar accuracies. Moreover, our proposed DPA is shown to be scalable with the number of computing nodes and capable of processing big hyperspectral images involving massive amounts of data.

## 1. Introduction

During recent years, hyperspectral remote sensing has been widely used in various fields of Earth observation and space exploration [1,2,3,4,5,6]. Hyperspectral sensors are able to simultaneously measure hundreds of contiguous spectral bands with fine spectral resolution [7]. In hyperspectral image (HSI) cubes, each pixel can be represented by a vector whose entries correspond to the spectral bands, providing a representative spectral signature of the underlying materials within the pixel [8,9,10,11,12,13]. This type of imagery offers detailed ground information in both the spectral and spatial domains. Therefore, HSIs have been popular in remote sensing applications such as urban mapping, environmental monitoring, object identification, and military defence [14,15,16,17,18].

Anomaly detection is one of the most popular and important techniques for distinguishing subtle differences among ground objects by exploiting the hundreds of narrow and nearly continuous bands provided by HSIs [19]. In fact, anomalous targets can be detected in HSIs without prior knowledge, i.e., the background refers to non-target pixels that are predominant in an image compared with the target pixels [20]. In the literature, many methods were proposed to improve the efficiency and effectiveness of hyperspectral anomaly detection [20,21,22,23,24,25,26,27,28,29]. An anomaly detection method based on low-rank and sparse representation (LRASR) was proposed in [30], which achieves robust anomaly detection with high accuracy, by taking full advantages of the correlations of all the pixels in HSIs and global information. However, LRASR involves a massive amount of matrix computations that are both computation-intensive and data-intensive, and becomes inapplicable to process big HSIs, since a single machine, even with powerful GPUs (note that GPUs have already been used to accelerate some serial algorithms [31,32,33]), can hardly provide sufficient computational resources. For instance, LRASR consumes 1.1 GB of memory when processing a small HSI with the size of 8000×162. However, it could not process a big HSI with the size of 800,000 ×162 on a single machine with 12 GB of memory, since all the memory is exhausted and the execution of LRASR is terminated with an “out of memory” exception, accordingly.

Distributed computing technologies are highly demanded for efficient processing of big HSIs. Cloud computing offers the potential to tackle massive data processing workloads by means of its distributed and parallel architecture. With the continuously increasing demand for massive data processing in HSI applications, there have been several efforts in the literature oriented towards exploiting cloud computing infrastructure for processing big HSIs [34,35,36,37,38,39,40]. Several parallel anomaly detection algorithms were proposed based on a master and slave model in [38,39,40] and executed on commodity clusters (or called public clouds). Based on the MapReduce parallel model [41,42,43,44,45], the approaches [34,35,36,37] use the Hadoop Distributed File System (HDFS) to implement distributed storage, and use Apache Spark [46] to execute HSI applications in parallel. Actually, Spark is an effective and state-of-the-art platform for parallel computation designed to efficiently deal with iterated computational procedures in terms of the MapReduce parallel model [47]. Spark employs HDFS to store the processed data in terms of blocks. The main abstraction of Spark is given by Resilient Distributed Datasets (RDDs), which represent a distributed, immutable, and fault-tolerant collection of objects [48]. When a task is executed on a computation node (called node for simplicity in this paper), all the data sets required by this need to be loaded into an RDD to construct a partition. In other words, a task corresponds to a partition. Meanwhile, users can also explicitly cache an RDD in memory across machines, and reuse it in multiple MapReduce parallel operations.

In this paper, we propose a novel distributed parallel algorithm (DPA) based on low-rank and sparse representations for hyperspectral anomaly detection. The proposed algorithm intends to accelerate LRASR on clouds. Independent computation operators in LRASR are first explored and executed in parallel on Spark. The hyperspectral data are reconstituted in an appropriate format for efficient DPA processing. The optimized storage is designed and a new pre-merge mechanism is developed for the purpose of reducing data transmission. Mean while, a new repartitioning policy is designed to improve DPA’s efficiency. Our experimental results show that the proposed DPA is able to achieve not only similarly good accuracies as LRASR but also very high speedups. Furthermore, DPA is verified to be scalable with the number of nodes and capable of processing big HSIs involving a massive amount of data.

The reminder of this paper is organized as follows. Section 2 gives a brief introduction to LRASR. Section 3 describes the newly proposed DPA, followed by the a presentation of experimental results in Section 4. Section 5 concludes with some remarks and hints at plausible future research lines.

## 2. Anomaly Detection Using Low-Rank and Sparse Representation (LRASR)

**Figure 1:**
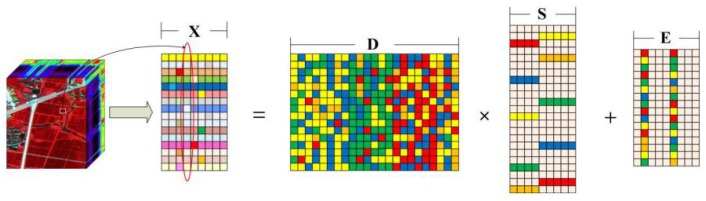
Data model of LRASR.

In a remotely sensed hyperspectral image, Anomalous pixels should be different from background pixels, while there usually exist strong correlation among background pixels. On this basis, our assumption is that background pixels can be linearly represented by some other background pixels, meaning that a HSI *X* can be decomposed into an anomalous part *E* and a background part D×S, where *D* is the background dictionary constructed from *m* background pixels, and *S* denotes the representation coefficients. In other words, we have X=DS+E. The data model of LRASR is shown in Figure 1 [30].

The math model of LRASR can be described using Equations (1) and (2), where ∥·∥ denotes the matrix nuclear norm (sum of singular values of a matrix), and ${∥\cdot ∥}_{1}$ is the $l_{1}$ norm of a matrix, i.e., the sum of the absolute value of all entries in the matrix. ${∥\cdot ∥}_{2,1}$ is the $l_{2,1}$ norm defined as the sum of $l_{2}$ norm of the column of a matrix. Besides, β>0 is a parameter to trade off low rankness and sparsity. λ>0 is used to balance the effects of the two parts.

(1) $$min_{S,E}{\{∥S∥}_{*}+{\beta ∥S∥}_{1}+{\lambda ∥E∥}_{2,1}\}$$

s.t.

(2) X=DS+E

**Figure 2:**
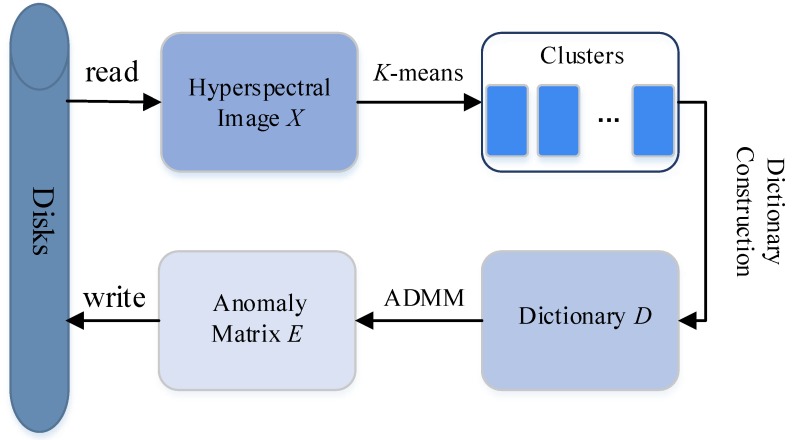
Flowchart of LRASR.

The algorithm LRASR was developed to solve the aforementioned problem, and its flowchart is given in Figure 2. From this figure, it can be seen that, after reading data from disks to obtain a HSI *X*, LRASR first employs the *K*-means algorithm to cluster all pixels, and then uses a dictionary construction method to obtain the background dictionary matrix *D* based on the obtained *K* clusters. Afterwards, an alternating direction multiplier method (ADMM) is used to generate the anomaly matrix *E*. Finally, the obtained anomaly matrix *E* is written to disks. Details of LRASR are given in Algorithm 1, in which Steps 1–16 denote the *K*-means algorithm, whereas Steps 17–24 represent the dictionary construction method. Step 25 is the ADMM, which is a complex algorithm and can be seen in [30].

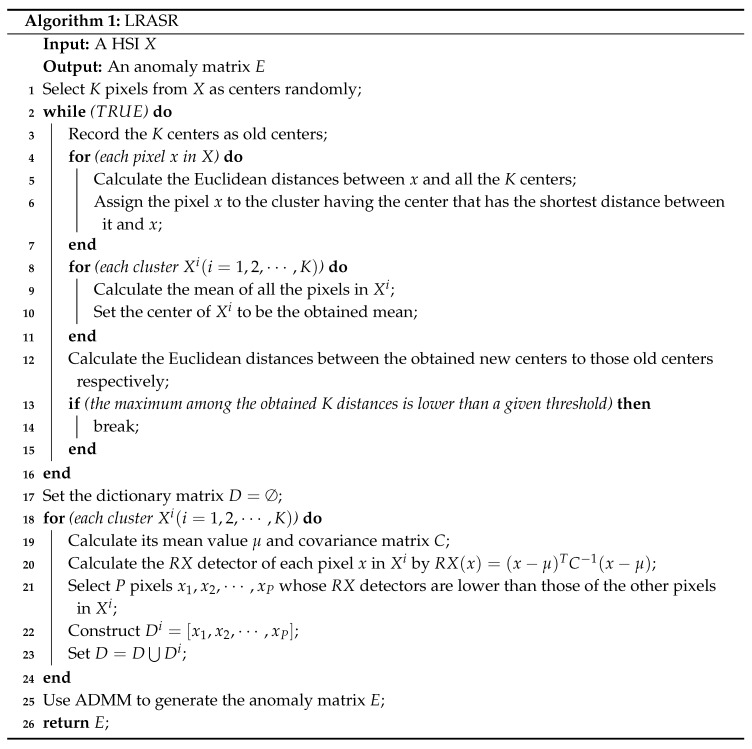



## 3. Distributed Parallel Algorithm (DPA)

**Figure 3:**
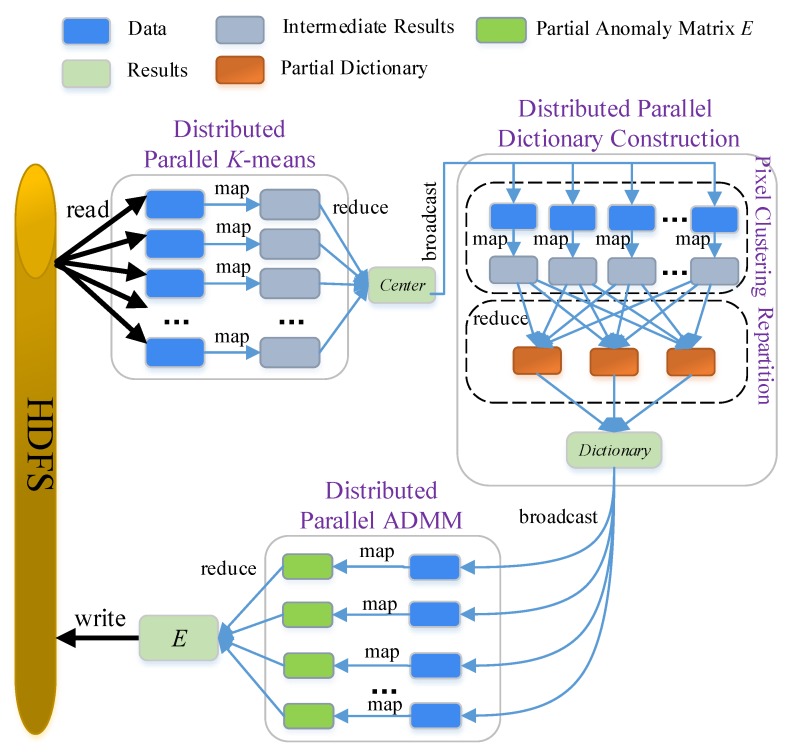
Flowchart of DPA.

Our newly developed DPA first explores the independent computation operators in LRASR and processes them in parallel on multiple nodes provided by Spark. In Algorithm 1, we can see that all the pixels are clustered by the *K*-means algorithm (Lines 1–16). In other words, the processing operator on each pixel, i.e., the calculation of Euclidean distance between the pixel and all *K* centers (Line 5) and the assignment of the pixel to a cluster whose center has the shortest distance to the pixel (Line 6), is not dependent on other pixels. Accordingly, the processing operators can be executed in parallel, and we propose a distributed parallel *K*-means algorithm to achieve this goal. Similarly, in the dictionary construction method (Lines 17–24), the processing operator on each cluster, including the calculation of the cluster’s mean value and covariance matrix (Line 19), the calculation of each pixel’s RX detector in the cluster (Line 20), the selection of *P* pixels from the cluster (Line 21) and the construction of $D^{i}$ (Line 22), is independent from other clusters. Accordingly, we establish a distributed parallel dictionary construction method, in which a new repartition policy is developed to use *K* nodes for the purpose of executing the processing operators of clusters in parallel. However, there is no independent computation operator in ADMM (details can be seen in [30]), since ADMM includes a loop in which each iteration depends on the previous one. Nevertheless, we construct a distributed parallel ADMM in which ADMM is conducted on each partition to generate a partial anomaly matrix, and a complete *E* can be obtained by composing all the partial matrices. The flowchart of our DPA is illustrated in Figure 3. Before detailing the three distributed parallel methods, we need to first describe data organization and storage optimization methods, which help the proposed DPA to reduce the data transmission and improve the efficiency.

### 3.1. Data Organization and Storage Optimization Methods

Band interleaved by line (BIL), band interleaved by pixel (BIP) and band sequential (BSQ) are three common formats for arranging HSIs’ data. BSQ stores bands sequentially, whereas BIL and BIP store bands in terms of lines and pixels, respectively. Being similar as LRASR, the proposed DPA processes pixels one by one. Accordingly, BIP is selected to organize HSIs’ data.

As the proposed DPA is executed in parallel on Spark, we need to upload HSIs’ data to HDFS storing data in blocks, whose size can be set according to different conditions and its default value is 64 MB. Given a program that can be divided into *m* computing tasks executed on *m* nodes in parallel (i.e., the level of parallelism is *m*), if the data required by one task are stored in multiple blocks on multiple nodes, all these blocks should be transferred to the node where this task is processed. Obviously, if a task’s required data are included in one block and the task is scheduled to the node storing the block, no data transmission is needed. Fortunately, Spark’s scheduler always targets an “optimized” assignment, i.e., assigning a task to a node where the task’s required data reside. Thus, a key aspect becomes how to make a task’s required data included in one block. This problem can be easily solved by setting the size of blocks to be d/m, where *d* is the total size of HSIs’ data. Note that for some special situations (e.g., the target node is extremely busy), Spark’s scheduler may fail to achieve the “optimized” assignment. When this failure occurs, data transmission is still needed. However, since the failure occurrence is in a relatively low probability, our proposed optimization method by determining the block size can still reduce data transmission.

From Figure 3, we can see that the HSIs’ data read from HDFS are used by all the three distributed parallel methods. For the purpose of fast data loading, these HSIs’ data are always maintained in memory after they are first read from HDFS.

### 3.2. Distributed Parallel K-Means Algorithm

**Figure 4:**
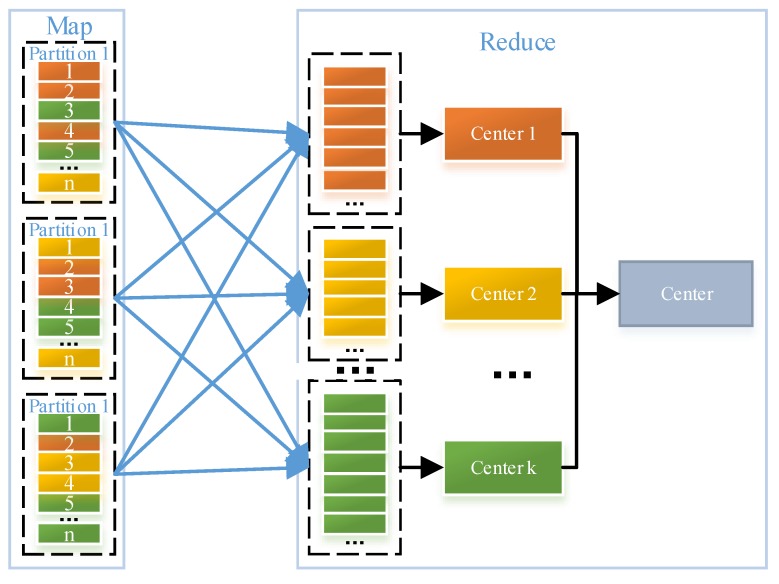
Data transmission before using the pre-merge mechanism.

The *K*-means algorithm shown by Lines 1–16 in Algorithm 1 contains three steps, which are described as follows: (1) calculate the Euclidean distances between each pixel and all the *K* centers (Line 5), and assign each pixel to a cluster with the center having the shortest distance to the pixel (Line 6); (2) for each cluster, obtain the mean of all the pixels in this cluster and set the cluster’s center to be the yielded mean (Lines 8–11); (3) check whether the termination criterion of the *K*-means algorithms is met (Lines 12–15). As mentioned before, the first step can be executed in parallel and is accordingly implemented as the Map method. Accordingly, as the second step needs all the pixels in each cluster to update each cluster’s center, and the third step needs all clusters’ new centers, both the two steps are implemented as the Reduce method. The algorithm outputs a center that is a set containing all obtained clusters’ centers. However, as shown in Figure 4, pixels belonging to the same cluster may appear in different nodes and need to be transferred to the Driver (i.e., a node where the Reduce method is executed). Alternatively, there would be much data transmission.

**Figure 5:**
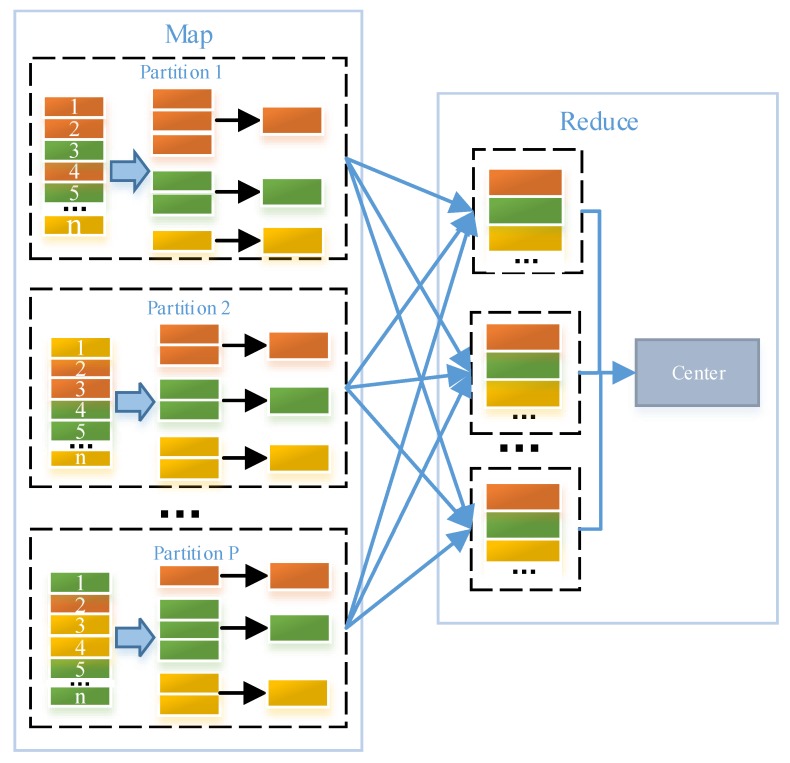
Data transmission after using the pre-merge mechanism.

A pre-merge mechanism is proposed to reduce the data transmission in our context. Before the data transmission, local pixels on each node are clustered using current clusters’ centers and then the sums of the pixels in each cluster are calculated. Rather than pixels, the obtained sums, the number of pixels involved to generate these sums and the corresponding clusters’ IDs that are necessarily required by the Reduce method are transferred from nodes where map methods are executed to the Driver. After all the map methods on multiple nodes are finished, the Reduce method on the Driver is able to calculate the mean of all the pixels in each cluster with these received data and update each cluster’s center. Afterwards, the Reduce method can check whether the termination criterion is met. Obviously, as the amount of transferred data (including the obtained sums, the number of pixels involved in generating these sums and the corresponding clusters’ IDs) is much lower than that of all the pixels, the pre-merge mechanism is able to reduce the data transmission. To clarify the pre-merge mechanism, we illustrate the data transmission after using the pre-merge mechanism in Figure 5. By comparing Figure 4 and Figure 5, we can observe that the pre-merge mechanism significantly contributes to the reduction of data transmission.

### 3.3. Distributed Parallel Dictionary Construction

According to LRASR, the background dictionary is constructed by composing the partial dictionary of each cluster obtained by the *K*-means algorithm. For each cluster, *P* pixels (whose RX scores are lower than those of the other pixels in the same cluster) are selected to generate the partial dictionary. In other words, the dictionary construction requires all the pixels of each cluster. However, the proposed distributed parallel *K*-means algorithm generates the *K* clusters without recording those pixels of each cluster. The reason is that, if we want to record pixels of each cluster, we need to write these pixels and their corresponding clusters’ IDs to RDD in every iteration. This procedure tries to write data into memory first, and then to disks (if there is not sufficient memory). As a result, this writing procedure consumes much memory and is time-consuming if insufficient memory exists, since writing data to disks is inefficient. To address this issue, we use a parallel pixel clustering operator implemented as the Map method (i.e., the pixel clustering phase in distributed parallel dictionary construction shown in Figure 3) which attributes all pixels to the *K* clusters. Afterwards, the partial dictionary construction is implemented as the Reduce method. As aforementioned, since the processing operator on each cluster (including the calculation of the cluster’s mean value and covariance matrix, the calculation of each pixel’s RX detector in the cluster, the selection of *P* pixels from the cluster and the construction of $D^{i}$) is independent from other clusters, we propose a repartition policy to employ *K* nodes to execute the processing operators of all the clusters in parallel. This procedure is quite different from the general one using the Driver only. Obviously, this repartition policy is able to improve DPA’s efficiency.

### 3.4. Distributed Parallel ADMM

As mentioned before, there are no independent computation operators in ADMM. Accordingly, we construct a distributed parallel ADMM, in which ADMM is conducted on each partition to generate a partial anomaly matrix, and a complete *E* can be obtained by composing all those partial matrices. As shown in Figure 3, these two procedures are implemented as the Map and the Reduce methods, respectively.

### 3.5. Comparison and Analysis

Compared with LRASR using three serial methods (i.e., the *K*-means algorithm, the dictionary construction method and ADMM), our proposed DPA employs three distributed parallel ones including the distributed parallel *K*-means algorithm, the distributed parallel dictionary construction method and the distributed parallel ADMM. Obviously, the advantage of our proposed DPA is that independent computation operators can be executed in parallel and HSIs’ data can also be processed in parallel.

We now analyze both the algorithms’ complexities. As our proposed DPA is derived from LRASR, we first calculate the complexity of LRASR and that of DPA can be obtained accordingly. Obviously, the complexity of the *K*-means algorithm is $O(WKT_{1})$, in which *W* is the total number of pixels, *K* denotes the number of centers and $T_{1}$ represents the total number of loops. The complexity of the dictionary construction method is $O(WP^{2})$ where *P* is the number of pixels selected from each cluster, whereas that of the ADMM is $O(PKT_{2}W^{2})$ where $T_{2}$ is the total number of loops. Let *m* be the number of nodes/data partitions. Accordingly, each node processes $\frac{W}{m}$ pixels. We can thus obtain the complexities of the distributed parallel *K*-means algorithm, the distributed parallel dictionary construction method and the distributed parallel ADMM are $O(\frac{WKT_{1}}{m})$, $O(\frac{WP^{2}}{m})$ and $O(\frac{PKT_{2}W^{2}}{m^{2}})$, respectively. In other words, the complexities of our proposed distributed parallel methods are $\frac{1}{m}$, $\frac{1}{m}$ and $\frac{1}{m^{2}}$ of those of the serial ones used by LRASR, respectively. Consequently, our proposed DPA is able to accelerate LRASR, remarkably.

## 4. Experimental Results

To verify the proposed DPA’s accuracy and efficiency, we perform four experiments on three Spark clusters (denoted as Spark1, Spark2 and Spark3) and six HSIs (called HSI1-HSI6). In the first two experiments (denoted as Experiment 1 and Experiment 2), HSI1 and HSI2 are processed by LRASR and our proposed DPA on Spark1, respectively. In the third experiment (regarded as Experiment 3), HSI1 is processed by LRASR and DPA on Spark2, separately. We design the experiments in this way to show DPA’s accuracy and efficiency when it is employed to process different HSIs on different platforms. Furthermore, in order to verify the DPA’s capability for processing big HSIs, we conduct the fourth experiment (denoted as Experiment 4), in which four HSIs (called HSI3-HSI6) with data sizes of 1 GB, 2 GB, 4 GB and 8 GB are generated by jointing HSI1 and used, respectively. HSI3-HSI6 are processed by DPA on Spark3, respectively. Details about the four experiments are summarized in Table 1.

Spark1 is a computing cluster which consists of 1 Master node and 8 Slave nodes. The Master is a virtual machine equipped with 4 cores (2G HZ) and 15 GB RAM. The Slaves are deployed on 4-blade IBM Blade Center HX5. Every Slave is configured with 6 cores (1.67G HZ) and 15 GB RAM. The Master and all the Slaves are installed with Ubuntu 16.04, Hadoop-2.7.3, Spark-2.1.1, Java 1.8 and Scala 2.11.6. As Spark considers each core in Slaves as a node, the total number of nodes is 48. In other words, the maximal level of parallelism of Spark1 is 48. Spark 2 includes 1 Master and 3 Slaves with identical configuration: 24 cores (2.3G HZ) and 64G of RAM. The Master and all the Slaves are installed with CentOS 6.6, Hadoop-2.7.3, Spark-2.1.1, Java 1.8 and Scala 2.11.7. The total number of nodes in Spark2 is 72, i.e., the maximal level of parallelism of Spark2 is 72. Spark3 is a very powerful cluster which consists of 1 Master and 6 Slaves. The Master and all the Slaves are virtual machines, each of which is equipped with 24 cores (2.5G HZ) and 242G RAM. The Master and all the Slaves are installed with Ubuntu 16.04, Hadoop-2.7.3, Spark-2.1.1, Java 1.8 and Scala 2.11.6. Accordingly, the total number of nodes in Spark3 is 144, i.e., the maximal level of parallelism of Spark3 is 144.

**Figure 6:**
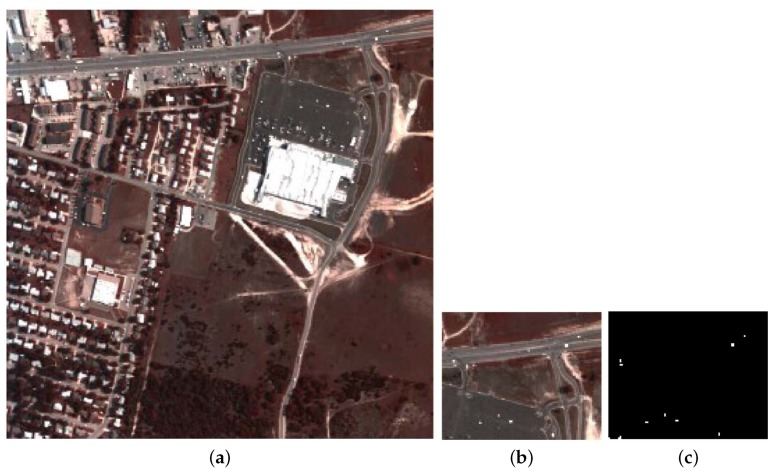
The first Hyperspectral Image (HSI1). (**a**) The false color image of the entire image; (**b**) The false color image of the chosen area for detection; (**c**) The ground-truth map of the chosen area [30].

Both HSI1 and HSI2 were used in [30]. HSI1 was collected by the hyperspectral data collection experiment (HYDICE) obtained from an aircraft platform. HSI1 covers an urban area (i.e., Fort Hood, TX, USA), comprising a vegetation area, a construction area, and several roads including some vehicles. A spectral resolution of 10 nm and a spatial resolution of 1 m are contained in this image. Multiple bands including the low-SNR and water vapor absorption bands (i.e., 1–4, 76, 87, 101–111, 136–153, and 198–210) are removed and 162 bands remain as a result. The entire image illustrated in Figure 6a has a size of 307×307 pixels, from which an area in the upper rightmost containing 80×100 pixels is selected for our experiments, since the ground truth shows that there are several anomalous targets (i.e., cars and roofs embedded in the different backgrounds) in this chosen area. A false color representation and the ground-truth map are given in Figure 6b,c, respectively.

**Figure 7:**
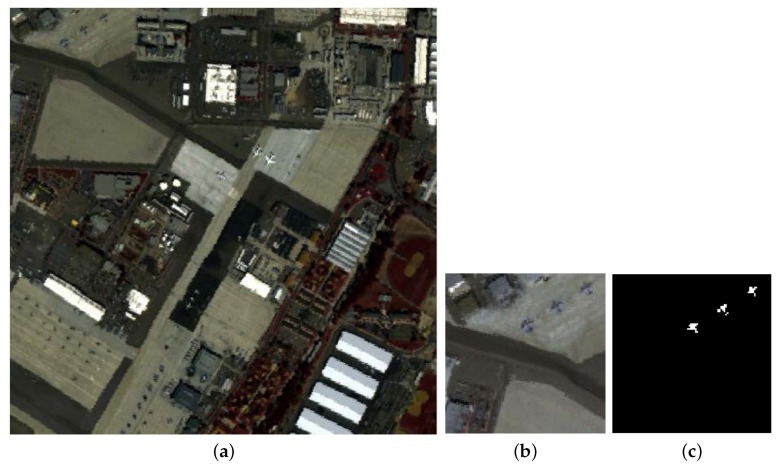
The second Hyperspectral Image (HSI2). (**a**) The false color image of the entire image; (**b**) The false color image of the chosen area for detection; (**c**) The ground-truth map of the chosen area [30].

HSI2 was collected by the Airborne Visible/Infrared Imaging Spectrometer (AVIRIS) over San Diego, CA, USA, comprising 224 bands in wavelengths ranging from 370 to 2510 nm. Some bands including low-SNR, water absorption and bad bands (i.e., 1–6, 33–35, 94–97, 107–113, 153–166, and 221–224) are removed. Consequently, 186 bands are left and used in our experiments. The entire image shown in Figure 7a has a size of 400×400, from which the up-left area including 100×100 pixels is selected to perform experiments. In this chosen area, there are buildings with different roofs, parking aprons with different materials, an airport runway, and a small quantity of vegetation. The airplanes are the anomalies to be detected. The false color image and the ground-truth map are illustrated in Figure 7b,c, respectively.

For the evaluation of accuracies and efficiencies of compared algorithms, AUCs (Area Under Curve) and consumed times of involved algorithms are recorded [49,50]. Larger AUCs indicate higher accuracies. In Experiments 1–3, LRASR is run on a single machine (i.e., the Driver node of each experiment corresponding Spark platform) since it is a serial algorithm, whereas DPA is run under different levels of parallelism evaluated by different numbers of nodes. The consumed times and AUCs of all the three experiments are recorded and shown in Table 2, in which consumed times are evaluated in seconds. On one hand, from Table 2, we can see that in Experiment 1, AUC of LRASR is 0.9181, whereas those of DPA with the numbers of nodes 2, 4, 8, 16 and 32 are 0.9202, 0.9184, 0.9131, 0.9195 and 0.9203, respectively. Meanwhile, we find that in Experiment 2, LRASR yields an AUC with the value of 0.9595, whereas DPA generates AUCs with the values of 0.9596, 0.9601, 0.9609, 0.9616 and 0.9607 when the numbers of nodes are 2, 4, 8, 16 and 32, respectively. Finally, in Experiment 3, LRASR obtains an AUC 0.9184, while DPA achieves AUCs 0.9218, 0.9141, 0.9188, 0.9116 and 0.9184 when the numbers of nodes are 2, 4, 8, 16 and 32, respectively. Please note that these AUCs in Experiment 3 are slightly different from those in Experiment 1. The reason is that the initial *K* centers are randomly selected from pixels in the *K*-means algorithm (see Line 1 in Algorithm 1). These results indicate that DPA achieves similarly good AUCs as LRASR, regardless of the level of parallelism.

On the other hand, in Experiment 1, we can see that LRASR consumes 3987 s, while DPA with the numbers of nodes 2, 4, 8, 16 and 32 consumes 1788 s, 955 s, 451 s, 233 s and 116 s. In Experiment 2, LRASR consumes 4957 s, whereas the consumed times of DPA are 2613 s, 1223 s, 586 s, 290 s and 149 s when the numbers of nodes are 2, 4, 8, 16 and 32, respectively. In Experiment 3, the times consumed by LRASR is 3657, while those consumed by DPA are 1699, 842, 418, 232 and 111, corresponding to the numbers of nodes 2, 4, 8, 16 and 32, respectively. These results indicate that our proposed DPA accelerates LRASR considerably in the premise of achieving similarly high accuracies. The reason is that, in our proposed DPA, independent computation operators are executed in parallel and all those data are also be processed in parallel.

**Figure 8:**
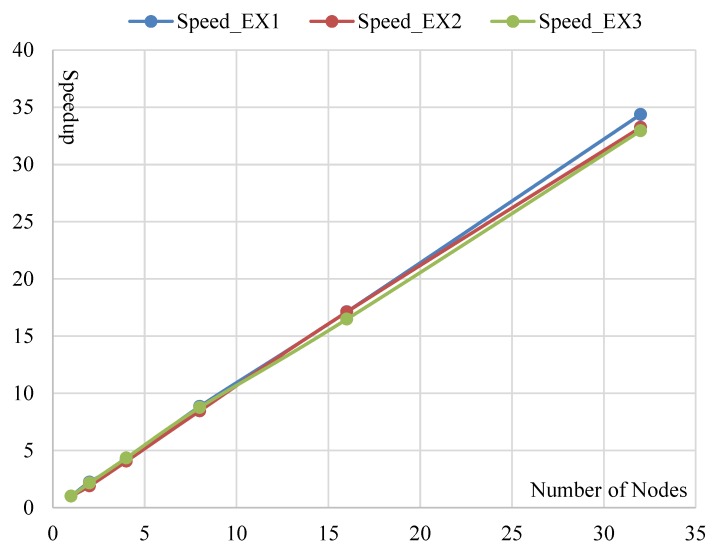
Speedups of DPA with different numbers of nodes in Experiments 1–3.

Based on those aforementioned consumed times, we can further calculate the speedups. The consumed times of LRASR is selected as the baseline. The result is illustrated in Figure 8. We can see that the speedups of DPA in Experiment 1 are 2.23, 4.17, 8.84, 17.11 and 34.37 when the numbers of nodes are 2, 4, 8, 16 and 32, respectively. This result indicates that the speedup of DPA increases linearly with the number of nodes. This conclusion illustrates the good scalability of the proposed DPA with the number of nodes. Meanwhile, the speedups of DPA are 2.23, 4.17, 8.84, 17.11 and 34.37 when the numbers of nodes are 2, 4, 8, 16 and 32, respectively. This result denotes that the speedups are larger than the numbers of nodes used for parallel computing, respectively. The reason is that, according to Section 3.5, the complexity of the serial ADMM is $O(PKT_{2}W^{2})$, whereas that of the distributed parallel ADMM is $O(\frac{PKT_{2}W^{2}}{m^{2}})$. In other words, the times consumed by the distributed parallel ADMM are $\frac{1}{m^{2}}$ of those consumed by the serial ADMM. Conclusively, the distributed parallel ADMM is able to achieve a remarkably high speedup.

For Experiment 2, it can be seen that the speedups of DPA are 1.9, 4.05, 8.46, 17.1 and 33.23 with the numbers of nodes 2, 4, 8, 16 and 32, respectively. Except the first speedup 1.9 is a little lower than its corresponding number of nodes 2, the others are larger than their corresponding numbers of nodes. The reason is the same as in Experiment 1. Meanwhile, we can get similar observations, i.e., the speedup of DPA increases linearly with the number of nodes and the distributed parallel ADMM obtains a good speedup. Furthermore, we can conclude that the proposed DPA achieves both good accuracies and speedups when processing different HSIs. For Experiment 3, we can see that the speedups of DPA are 2.15, 4.34, 8.75, 16.47 and 32.94 with the numbers of nodes 2, 4, 8, 16 and 32, respectively. By comparing this result with that of Experiment 1, we can obtain similar conclusions. In other words, both high accuracies and speedups can be achieved by the proposed DPA running on different platforms.

**Figure 9:**
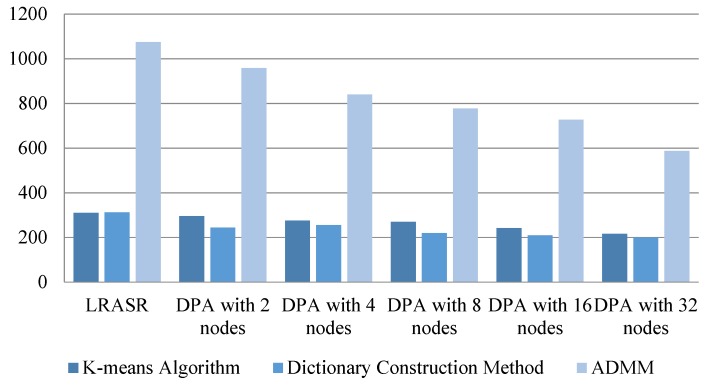
Memory consumption (MB) of LRASR and DPA with different number of nodes in Experiment 1.

To show the advantage of our proposed DPA on memory consumption, the memory consumption of LRASR and DPA with different number of nodes in Experiment 1 is illustrated in Figure 9, in which the mean amounts of memory consumed by the three methods (i.e., the *K*-means algorithm, the dictionary construction method and the ADMM) of LRASR and DPA on all involved nodes are recorded. Please note that the three methods of LRASR are serial ones, whereas those of DPA are distributed parallel ones. Figure 9 indicates that the mean amounts of consumed memory of DPA’s methods are lower than those of LRASR’s methods, without respect of the DPA’s parallelism, respectively. Meanwhile, we can also see that the mean amounts of consumed memory of DPA’s methods are reduced while the number of nodes increases. These conclusions imply that our proposed DPA could process big HSIs, especially when DPA is executed in high parallelism.

**Figure 10:**
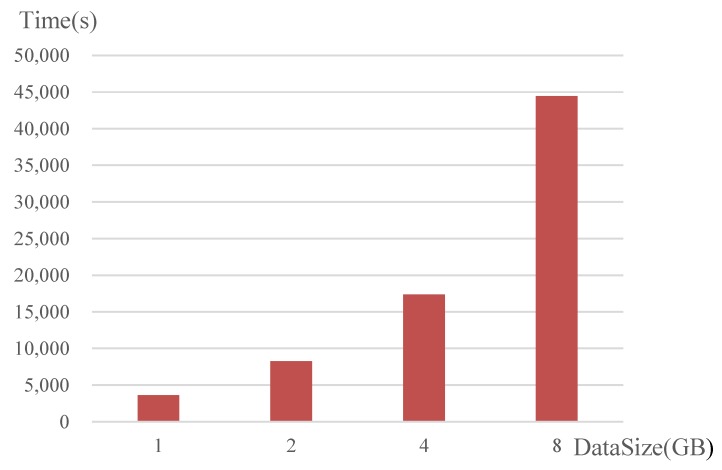
Speedups of DPA processing big HSIs with different data sizes.

To verify the capability of the proposed DPA to process big HSIs with massive amounts of data, we perform Experiment 4, in which the level of parallelism is fixed to a large value 120. The results are shown in Figure 10, which shows that the consumed times are 3603, 8284, 17,367 and 44,421 s for HSI3–HSI6, respectively. The experimental results indicate that, by means of distributed parallel computing, the proposed DPA is able to efficiently process the big HSIs, which cannot be processed by LRASR running on a single computing machine with limited resources.

To show more insights, we compare the results of LRASR and our proposed DPA running on one node. In other words, DPA is running serially and denoted as SDPA, accordingly. Both of the two compared algorithms are executed using HSI1 on the Driver node of Spark1. The experimental results show that SDPA achieves an AUC 0.9197 and consumes 4320 s. On one hand, compared with the LRASR’s AUC 0.9181, SDPA’s AUC is similarly high. On the other hand, by comparison with the LRASR’s consumed times 3987 s, it can be seen that SDPA requires more computation times. The reason is that SDPA is actually a parallel-designed algorithm in terms of the MapReduce model. Accordingly, although it is serially executed on a single node, there will also be much intermediate data generated and written to the shared memory by map methods, and afterwards, these intermediate data will be loaded by the reduce method from the shared memory. These overheads, including data generation and data written/loaded to/from shared memory, reduce the efficiency. However, DPA achieves good speedups when it is executed in parallel on multiple nodes. Consequently, we can conclude that Spark is appropriate to process parallel-designed algorithm in parallel rather than in serial.

Furthermore, we re-implement DPA and execute the obtained algorithm (denoted as HDPA) on Hadoop that is similar as Spark, i.e., both of them are able to execute MapReduce-based applications. The main difference between them is that Spark supports in-memory computation while Hadoop does not. In this experiment, HSI1 is used and Hadoop is deployed on the same cluster as Spark1. The HDPA’s results (including AUCs and consumed times) are given in Table 3. By comparing these results with those of DPA in Experiment 1 (seen in Table 2), we can observe that both HDPA and DPA achieve similarly good AUCs, whereas DPA outperforms HDPA in terms of consumed times for all the cases with different number of nodes. This conclusion indicates that Spark is more effective than Hadoop due to its in-memory computation capability.

## 5. Conclusions and Future Work

With the ever growing size and dimensionality of HSIs, it is a challenge to perform anomaly detection in big hyperspectral images on a single computing machine. This paper introduces a novel DPA algorithm to accelerate an LRASR method for hyperspectral anomaly detection on cloud computing architectures. The newly developed algorithm presents several important contributions. First, independent computation operators are explored and executed in parallel on Apache Spark, which is a promising distributed computing platform to process massive data in parallel. In addition, the hyperspectral data are reconstituted in an appropriate format for efficient DPA processing. Meanwhile, a new pre-merge mechanism has been developed for further reducing data transmission, and a new repartitioning policy was used to improve DPA’s efficiency. We evaluated the accuracy and efficiency of our newly developed DPA by means of multiple experiments. The experimental results demonstrated that DPA not only obtained similarly high accuracies as LRASR, but also achieved considerably higher speedups. Moreover, DPA was also verified to be scalable with the number of nodes and capable of processing big HSIs with massive amounts of data. These conclusions indicate that the Spark is very appropriate for processing HSIs’ applications that are both computation-intensive and data-intensive (e.g., LRASR). As massive amounts of intermediate data would be generated and transferred among nodes, we need to design some optimized strategies (e.g., our proposed pre-merge mechanism in the distributed parallel *K*-means algorithm) to reduce data transmission. In future work, we will develop cloud implementation of other HSI processing algorithms, including techniques for classification and unmixing.

## Figures and Tables

**Table 1:** Details about the four experiments.

Experiments	HSIs	Platforms
Experiment 1	HSI1	Spark1
Experiment 2	HSI2	Spark1
Experiment 3	HSI1	Spark2
Experiment 4	HSI3-HSI6	Spark3

**Table 2:** AUCs and Consumed Times(s) obtained by LRASR and DPA with different numbers of nodes in Experiments 1–3.

Number of Nodes	Times (EX1)	AUC (EX1)	Times (EX2)	AUC (EX2)	Times (EX3)	AUC (EX3)
LRASR	3987	0.9181	4957	0.9595	3657	0.9184
DPA with 2 Nodes	1788	0.9202	2613	0.9596	1699	0.9218
DPA with 4 Nodes	955	0.9184	1223	0.9601	842	0.9141
DPA with 8 Nodes	451	0.9193	586	0.9609	418	0.9188
DPA with 16 Nodes	233	0.9195	290	0.9616	232	0.9116
DPA with 32 Nodes	116	0.9203	149	0.9607	111	0.9184

**Table 3:** AUCs and Consumed Times(s) obtained by HDPA with different numbers of nodes.

Metrics	2 Nodes	4 Nodes	8 Nodes	16 Nodes	32 Nodes
AUC	0.9216	0.9166	0.9187	0.9141	0.9155
Times	4320	2524	2272	1706	1706
